# Validation of the Mediating Effect of Parenting Stress in the Relationship between Work-Family Strains and Gains and Marital Satisfaction for Fathers in Korea

**DOI:** 10.3390/children11101170

**Published:** 2024-09-26

**Authors:** Si-Young Lee, Hye-Sun Jung

**Affiliations:** 1 Department of Public Health, Graduate School, The Catholic University of Korea, Seoul 06591, Republic of Korea; bohemianevan@naver.com; 2 Department of Preventive Medicine, College of Medicine, The Catholic University of Korea, Seoul 06591, Republic of Korea

**Keywords:** father, work-family strains, work-family gains, marital satisfaction, parenting stress, partial mediation

## Abstract

Background/Objectives: This study aimed to examine the relationship between Korean fathers’ work-family strains and gains and marital satisfaction and to validate the mediating effect of parenting stress in the relationship. Methods: This study used the 14th-year survey data of the Panel Study of Korean Children (PSKC) conducted by the Korea Institute of Child Care and Education (KICCE), the latest available data. The PSKC is a longitudinal study of children’s growth and development from birth in the Republic of Korea. After excluding datasets containing missing values from the data collected from 2150 respondents, the data were extracted from the survey responses collected from 1130 respondents, and descriptive statistical analysis, path analysis, and mediating effect analysis were conducted. Results: First, Korean fathers’ work-family strains had a positive (+) influence on parenting stress (β = 0.658, *p* < 0.001). Second, Korean fathers’ work-family strains had a negative (−) influence on marital satisfaction (β = −0.185, *p* < 0.001). Third, Korean fathers’ work-family gains did not have a statistically significant influence on parenting stress (β = 0.016, *p* = 0.632). Fourth, Korean fathers’ work-family gains had a positive (+) influence on marital satisfaction (β = 0.381, *p* < 0.001). Fifth, Korean fathers’ parenting stress did not have a statistically significant influence on marital satisfaction (β = 0.011, *p* = 0.783). Sixth, the indirect effect of work-family strains on marital satisfaction via parenting stress (β = 0.013, 95% Bias-corrected CI = −0.042∼0.075) was statistically significant, and specifically, it had a partial mediating effect. Seventh, the indirect effect of work-family gains on marital satisfaction via parenting stress (β = 0.028, 95% Bias-corrected CI = 0.005∼0.051) was statistically significant, and specifically, it had a partial mediating effect. Conclusions: This study aims to provide the basic data for various family support policies for fathers by understanding the current status of work-family balance for fathers in modern society, as well as by examining the effects of parenting stress in the relationship with marital satisfaction.

## 1. Introduction

Since recording 1.68 children per woman in 2016, the average fertility rate among 38 member countries of the Organization for Economic Cooperation and Development (OCED) has consistently declined to reach 1.49 children per woman in 2022 [1]. Among the countries, Korea, in particular, was the only OECD country in 2018 to report a fertility rate of less than one child per woman [2]. This declining trend has continued since then to reach 0.72 children per woman as of 2023, which is the lowest figure reported after the country’s fertility rate database was first established in 1970 [3].

Aside from a mere decrease in population, low fertility rates can have various economic impacts, including a decline in the share of the working-age population out of the total population and a consequent increase in expenses to support the aged population; low fertility rates could also slow down economic growth caused by increased uncertainty surrounding future incomes [4]. The steady increase in the aged population is also expected to result in an increase in social problems such as the issue of caring for senior citizens [5]. While the potential for environmental benefits has been discussed [6], the international community understands the issue of the declining birthrate as a common challenge and is focusing on finding a solution.

In the early stage of examining the cause of the low fertility rate issue, research findings presented across various research areas and target groups indicated that the predominant empirical factor for low fertility rates was the increase in the number of women with a higher level of education [7]. Recently, however, the rebound in fertility rates in the 21st century, after hitting the lowest level in the late 20th century, has been observed to be closely associated with gender equity policies or work-family policies [8]. What is related to low fertility rates is not gender equity within the individual-oriented system—meaning equity between men and women in areas such as education, employment, and health—but gender equity within the family-oriented system, referring to equity between husband and wife in the marital relationship [9]. Considering these trends in previous studies, this study assumed that the cause of declining fertility rates is associated with marital satisfaction.

Marital satisfaction refers to the subjective assessment of the quality of marital life by a man and a woman in a marital relationship; the overall feelings and emotions that individuals have concerning marriage not only affect the psychological satisfaction of the husband and wife but also the growth of children, the functioning of the family, as well as social stability [10]. In this regard, despite the need for profound research on the marital satisfaction of married men and women that is required, most previous studies have focused on the marital satisfaction of only married women or mothers [11,12]. As industrialization advanced, an increasing number of women supposedly began to participate in economic activities. This led to conflicts with the traditionally expected gender roles, consequently resulting in changes in marital satisfaction. However, in modern society, working for prolonged hours is still socially acceptable for men, while their roles and responsibilities in the family are also increasing. In this context, the marital satisfaction of men is gaining attention as an important factor in the family and society. Thus, some recent studies have examined the effect of gender on marital satisfaction [13], once again emphasizing the need to understand the difference between genders in the perception of work-family strains by gender role norms [14].

The daily life of married adults is typically divided into the two categories of work life and family life; thus, work-family balance is a frequently examined research topic along with the marital satisfaction of married couples. The two domains of work-life balance are known to be independent of each other, but a high level of association exists between them. They establish a close relationship where positive or negative experiences in one domain affect the other [15]. Among them, assuming restrictions on the time and energy consumed by an individual [16], work-life strains indicate the inevitable tension generated by the two roles performed by the individual as a worker and as a family member [17]. Contrarily, work-family gains originated from the alternative concept that performing multiple roles improves social support, interactions, and the development of skills, whose benefits offset the disadvantages despite time constraints [18]. In general, women reportedly feel more strain when they spend more time performing the role of a worker than performing roles within the family, while men feel more strain when their work life is significantly interfered with by family life [19]. However, gender differences in work-life strains could vary from culture to culture [20]. In Korea, studies on the work-family balance of fathers have been mostly conducted on work-related variables such as job satisfaction [21,22], and job involvement [23]. However, a new approach is required as the goal of work-life balance has shifted from achieving a successful work life to maintaining a happy family life, reflecting changing interests among fathers in line with social changes.

In the relationship between work-life balance and marital satisfaction, to investigate the fundamental cause of the issue of low fertility rate faced by Korean society, the present study intended to examine and validate the mediating effect of parenting stress, regarded as the most serious challenge in childbirth and child-rearing, on the relationship between Korean fathers’ work-family strains and gains and marital satisfaction. Parenting stress indicates the psychological burdens and tension felt by parents owning to the burdens and responsibilities related to raising children and performing their parental roles [24]. Stress factors arising from parenting, such as learning and taking on new roles as parents, excessive pressure from parental responsibilities, and time constraints, reportedly reduce positive interactions and increase negative communication between the husband and wife; this makes the couple inappropriately respond to conflicts within the family, consequently resulting in reduced marital satisfaction [25]. As parenting stress is expressed in various forms influenced by factors such as country [26,27], culture [28], and the environment of parents or children [29], assessing and evaluating parenting stress from a single perspective is difficult. It requires a comprehensive understanding that takes these factors into account. For instance, one of the representative characteristics of parenting stress in Korea is the high interest in and expectations regarding the academic performance of the child, which is caused by the fierce competition in Korea to enter elite universities. Korean parents reportedly feel parenting stress when their expectations regarding their children’s academic performance are not met in reality [30]. Moreover, even when parents’ expectations are met with high academic performance from their child, many other factors also affect family life in diverse aspects, such as educating children earlier than the normal curriculum regardless of their developmental status, which could negatively impact children’s development, or increased expenses for private tutoring and other private educational programs, which could impose an additional burden on the family budget. In the findings of previous studies conducted abroad on parenting stress, parenting stress was observed to be strongly correlated with a decline in the marital satisfaction of married couples, defined as the subjective assessment of the overall quality of marital life [31]. Furthermore, parenting was found to affect the decrease in marital satisfaction of the couple due to the dramatic increase in housework hours and the decrease in the amount of time spent with the partner [32]. However, many of these previous studies used mothers’ reporting as a tool to measure interactions between parents and their children, and only a few studies investigated father-child interactions, although the quality of such father-child interactions could have an impact on the child [33]. In this regard, there is an increasing need for research on fathers.

Regarding this study, a review of previous studies showed that as tension intensifies between the assumption that an ideal worker is not restricted by personal life and the changing image of an ideal father, men could encounter similar struggles to those faced by women [34]. Several studies have also focused on the impact of fathers’ parenting stress and depression on family [35,36,37,38,39]. Moreover, in Korea, with increasing work-family imbalance for men, the marital relationship was found to become more negative, resulting in increased conflicts [40]. Fathers reportedly feel more parenting stress when they are better aware of such conflicts in co-parenting [41], and they have a higher level of parenting stress when they face more conflicts and challenges in the process of maintaining a balance between work and family [42]. In the previous studies mentioned above, although significant findings were presented regarding parenting stress, the influence of parenting stress on work-family strains and gains was not evaluated, and the studies did not simultaneously explore marital satisfaction and parenting stress from a structural perspective. With the dramatic social changes due to the COVID-19 pandemic, such as the spread of remote working, fathers are no longer faced with the increasing expectations of “direct”, “participatory”, and “friendly” fathers [43]. Thus, a new perspective is required to investigate this trend. Consequently, this study aimed to investigate the relationship between Korean fathers’ work-family strains and gains and marital satisfaction. Furthermore, it aimed to validate the mediating effect of parenting stress in that relationship.

**Figure 1:**
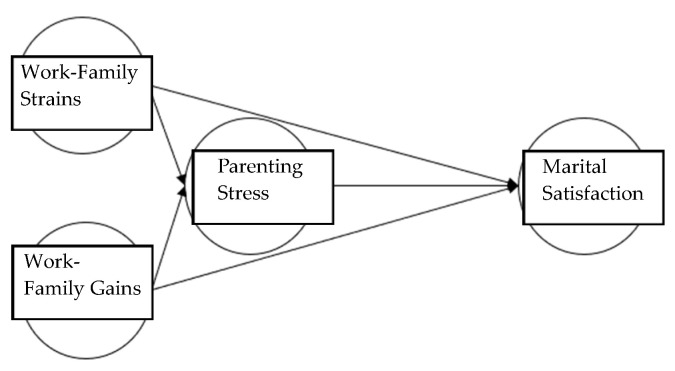
Hypothesis model.

**Figure 2:**
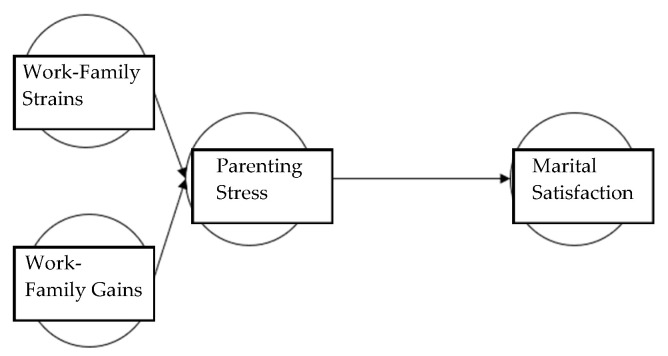
Competition model.

For the purpose of this research, this study suggested the following research hypotheses and research models (Figure 1 and Figure 2).

First, for fathers in Korea, work-family strains have a positive (+) influence on parenting stress. Second, for fathers in Korea, work-family strains have a negative (−) influence on marital satisfaction. Third, for fathers in Korea, work-family gains have a negative (−) influence on parenting stress. Fourth, Korean fathers’ work-family gains have a positive (+) influence on marital satisfaction. Fifth, Korean fathers’ parenting stress has a negative (−) influence on marital satisfaction. Sixth, in the relationship between Korean fathers’ work-family strains and marital satisfaction, parenting stress has a mediating effect. Seventh, in the relationship between Korean fathers’ work-family gains and marital satisfaction, parenting stress has a mediating effect. This study attempted to validate the mediating effect through a comparison between the hypothesis model (full mediation model, Figure 1) and the competition model (partial mediation model, Figure 2).

## 2. Materials and Methods

### 2.1. Participants

This study aimed to validate the mediating effect of parenting stress in the relationship between fathers’ work-family gains and strains and marital satisfaction. For the purpose of this study, the 14th-year survey data of the Panel Study on Korean Children (PSKC), which was conducted by the Korea Institute of Child Care and Education (KICCE) in 2021, were analyzed.

The first survey for the PSKC was conducted in 2008 for a long-term longitudinal study of children in Korea from birth. The objective was to collect and provide longitudinal data at the national level on the growth and developmental characteristics of children for individual periods, parenting status and demands of parents, functions and effects of childcare supporting institutions, and roles and effects of local communities and childcare policies. Since then, 14 surveys have been conducted to provide a wide range of basic data for the characteristics of children, parents, families, schools, childcare supporting services, local communities, and childcare supporting policies. From the 2021 PSKC survey data, the data of 2150 respondents are currently available (https://panel.kicce.re.kr/pskc/module/rawDataManage/index.do?menu_idx=56 accessed on 23 September 2024). From the available data, 985 who said they were mothers or other carers and 35 datasets with missing values were excluded. Only those of 1130 fathers who had given birth to a baby in 2008 and have been rearing the child were selected and analyzed.

This study was approved for the institutional review board review exemption (MC24ZASI0038) by the Institutional Review Board of the Catholic University of Korea.

### 2.2. Research Instruments

#### 2.2.1. General Characteristics of This Research Participants

The general characteristics of these research participants are presented in Table 1. In terms of age, people in their 40s made up the largest share with 879 respondents (77.79%), followed by those in their 50s with 220 respondents (19.47%) and those in their 30s with 31 respondents (2.74%). When asked whether they were living with their child, 1072 respondents (94.87%) reported that they were, while 58 respondents (5.13%) said they were not. Regarding marital status, 1110 respondents (98.23%) were married, and 20 respondents (1.77%) were not married but raising the child. For educational background, 698 respondents (61.77%) had a bachelor’s degree, followed by 290 high school graduates or those with a lower level of education (25.66%) and 142 respondents (12.57%) with a master’s degree.

#### 2.2.2. Fathers’ Work-Family Strains and Gains

To measure fathers’ work-family strains and gains, the PSKC translated and used 26 items developed by Marshall and Barnett in 1993 [44]. For the measurement, each item was structured on a 5-point Likert scale (absolutely incorrect = 1, totally correct = 5). In the panel data, the 26 items on “fathers’ work-family strains and gains” were divided into 15 items on work-family strains and 11 items on work-family gains, but any sub-variables concerning each of the strains and gains were not presented separately. In utilizing the data for this study, exploratory factor analysis was conducted for work-family strains and work-family gains. The exploratory factor analysis results for the items on work-family strains are presented in Table 2.

According to the exploratory factor analysis results of the items on work-family strains, items 1, 5, 6, 7, 8, and 9 were identified as one subfactor, and the subfactor was labeled as “time constraints”. Items 11, 12, 13, 14, and 15, which were identified as another subfactor, were labeled as “decline in work performance”, while the remaining items 2, 3, 4, and 10 were categorized as the subfactor labeled as “role as a parent”. The Kaiser–Meyer–Olkin (KMO) value was 0.947, Bartlett’s χ^2^ was 10,402.604, and the *p*-value was < 0.001.

The results of the exploratory factor analysis of work-family gains are presented in Table 3.

Among the items on work-family gains, items 8, 9, 10, and 11 were classified as one subfactor, labeled as “maintaining identity as an individual”. The subfactor consisting of items 4, 5, 6, and 7 was labeled as “self-management”, while the subfactor comprising the remaining items 1, 2, and 3 was labeled as “role as a parent”. The KMO value was 0.915, Bartlett’s χ^2^ was 8655.649, and the *p*-value was < 0.001.

#### 2.2.3. Marital Satisfaction

Marital satisfaction was measured using the Revised Kansas Marital Satisfaction Scale, which was revised by Chung in 2004 [45] from the Kansas Marital Satisfaction Scale by Schumm et al. in 1983 [46] considering the Korean culture. The revised measuring tool, which was based on a 7-point scale, was once again modified to be based on a 5-point scale in the Panel Study on Korean Children. The tool included items on satisfaction with the wife as a spouse, satisfaction with marital life, satisfaction with the relationship with life, and satisfaction with the wife as the mother of the child. For marital satisfaction, explorative factor analysis was not conducted separately as each item was considered a sub-variable.

#### 2.2.4. Parenting Stress

Parenting stress was measured using the measuring tool developed by Kang in 2003 [47]. It consists of 24 items. Among those items, 7 items that were not appropriate for the age of this study participants were excluded, and only the remaining 17 items were employed for measurement. When measured on a 5-point Likert scale, higher scores mean a higher level of parenting stress. As sub-factors were not presented separately, exploratory factor analysis was conducted for the purpose of this study, and the analysis results are presented in Table 4.

According to the exploratory factor analysis results of items on parenting stress, items 1, 2, 3, 4, 5, and 6, items 7, 9, 10, 11, 12, 14, 15, and 16, and items 8, 13, and 17 were categorized into three sub-factors, and each of the sub-factors was labeled as “school variable”, “education and learning”, and “aptitude and growth”, respectively. The KMO value was 0.946, Bartlett’s χ^2^ was 12,298.254, and the *p*-value was <0.001.

### 2.3. Reliability and Validity of Research Instruments

The PSKC data did not present validity values by instrument, but this research instrument profile was provided so that individual researchers could calculate validity. For this reason, this study conducted reliability analysis by variable to calculate Cronbach’s α. The reliability of each variable used in this study is presented in Table 5.

The reliability of sub-variables was between 0.728 and 0.935. As a value of 0.7 or higher is generally considered appropriate [48], this study concluded that each research instrument had an appropriate level of reliability.

In the following step, confirmatory factor analysis was conducted to test the validity of this research model, and the model fit was as presented in Table 6. Specifically, the χ^2^/DF value was 5.914, the root mean square residual was 0.024, the goodness of fit index was 0.954, the normed fit index was 0.966, the incremental fit index was 0.972, the comparative fit index was 0.972, and the root mean square error of approximation was 0.066, all of which were acceptable. In particular, as the comparative fit index and the root mean square error of approximation were presented as important in this study by Hong in 2000 [49], this study utilized those values as the criteria for judgment.

The confirmatory factor analysis results are as presented in Table 7. For all of the variables, the standardized regression coefficient was between 0.697 and 0.930, while significance (critical ratio) was observed to be 1.965 or more. Construct reliability for each variable was between 0.886 and 0.959, while the average variance extracted was between 0.724 and 0.853, which showed that convergent validity was secured.

Next, discriminant validity was tested. The strictest method to test discriminant validity is to compare the squared value of the correlation coefficients of the two variables with the highest correlation coefficients and the average variance extracted value of those variables. As presented in Table 8, the squared value of the correlation coefficient for “parenting stress ↔ work-family strains”, which showed the highest correlation coefficient”, was 0.282, which was lower than the average variance extracted values of parenting stress (0.724) and work-family strains (0.840), confirming that discriminant validity was secured.

### 2.4. Data Collection and Analysis

This study used the 14th-year PSKC data, which is the latest available data. The data were collected using a collection method combining paper and pencil personal interviews, computer-assisted personal interviews, and tablet-assisted personal interviews from 1165 fathers. The data from 35 respondents were excluded for containing incomplete responses. Consequently, data from 1130 respondents were analyzed in this study utilizing SPSS and the AMOS 23 program (IBM Corp., Armonk, NY, USA). The details of the analysis results are as follows. First, frequency analysis was performed to examine the general characteristics of this study participants (Table 1). Second, exploratory factor analysis was conducted to classify the subfactors of each latent variable (Table 2, Table 3 and Table 4). Third, the reliability of this research instruments used in this study was tested through Cronbach’s α (Table 5). Fourth, through confirmation factor analysis of this research model, estimates and model fit were assessed (Table 6, Table 7 and Table 8). Fifth, descriptive statistical analysis was carried out to investigate the participants’ perceptions of each variable (Table 9). Sixth, a path analysis of the structural relationship among validated variables was conducted (Table 10). Seventh, to validate the mediating effect of parenting stress in the relationship between work-family gains and strains and marital satisfaction, effects were categorized into direct, indirect, and total effects, and the statistical significance of the indirect effect was calculated through bootstrapping. In consideration of this study results by Shrout and Bolger (2002) [50], it could not be concluded that the mediating effects showed stationarity. Consequently, 1000 bootstrapping sample datasets, generated from the raw data through random sampling, were suggested for use for parameter estimation, with the confidence interval set at 95%. This study followed the suggestion as is. In addition, this study investigated the indirect effect through which work-family strains and gains proceed via parenting stress to marital satisfaction.

## 3. Results

### 3.1. Descriptive Statistical Analysis

Descriptive statistical analysis was conducted for all of the variables used in this study (work-family gains, work-family strains, marital satisfaction, and parenting stress) and their sub-variables, and the analysis results are presented in Table 9. Mean values were distributed between 2.11 and 4.02, while the standard deviation was distributed between 0.65 and 0.88. According to skewness and kurtosis values, absolute skewness values were distributed between 0.033 and 0.746, while absolute kurtosis values were distributed between 0.054 and 1.261. These results were considered to satisfy the requirements for normality of the structural equation model.

### 3.2. Path Analysis

The proposed model established in this study consists of four latent variables—work-family gains, work-family strains, marital satisfaction, and parenting stress. Additionally, it contains 13 measured variables: maintaining an individual’s identity gains, self-management gains, parental role gains, time constraints strains, decline in work performance strains, parent’s role strains, parenting stress from school variables, parenting stress from education and learning, parenting stress from aptitude and growth, satisfaction with spouse, satisfaction with marital life, satisfaction with relation, and satisfaction with motherhood. As presented in Table 10, the results showed that the value calculated by dividing χ^2^ by the degree of freedom was 5.914, the root mean square residual was 0.024, the goodness of fit index was 0.954, the normed fit index was 0.966, the incremental fit index and the comparative fit index were 0.972, and the root mean square error of approximation was 0.066, indicating that the fit index satisfied the criteria.

Next, the path analysis results are as presented in Table 11. Specifically, first, Korean fathers’ work-family strains were observed to have a positive (+) influence on parenting stress (standardized regression coefficient = 0.658, *p* < 0.001). Second, Korean fathers’ work-family strains were observed to have a negative (−) influence on marital satisfaction (standardized regression coefficient = −0.185, *p* < 0.001). Third, Korean fathers’ work-family gains did not have a statistically significant influence on parenting stress. Fourth, Korean fathers’ work-family gains were observed to have a positive (+) influence on marital satisfaction (standardized regression coefficient = 0.381, *p* < 0.001). Fifth, Korean fathers’ parenting stress did not have a statistically significant influence on marital satisfaction.

### 3.3. Mediating Effect

To validate the mediating effect of parenting stress in the relationship between Korean fathers’ work-family strains and gains and marital satisfaction, analysis was conducted to test a model that could provide a desirable explanation for the structural relationship of each variable. The results were as follows: First, an analysis and comparison of model fit were performed between the partial mediation model, where work-family strains and gains go through parenting stress and simultaneously have direct effects on marital satisfaction (hypothesis model), and the full mediation model, where there is no direct path between work-family strains and gains and marital satisfaction, and work-family strains and gains affect marital satisfaction via parenting stress (competition model). Next, the most simple and suitable model that could best explain the experiential data were identified. Moreover, as presented in Table 12 and Table 13, the model fit index was calculated to compare between the hypothesis and competition models.

As the full mediation model is a latent model contained in the partial mediation model, χ^2^ difference test was performed. In the χ^2^ test with the degree of freedom level at 0.05, the difference value should be 5.99 when the goodness of fit of the two models is statistically significant. The χ^2^ difference between the hypothesis and competition models for work-family strains was 75.076, and the difference in the degree of freedom was 1. For work-family gains, the χ^2^ difference between the hypothesis and competition models was 182.02, and the difference in degree of freedom was 1, showing a statistically significant difference at the level of α = 0.05. As the partial mediation model is accepted when the χ^2^ difference test results are statistically significant and the full mediation model is accepted when those results are not statistically significant [49], the partial mediation model, which is the competition model for work-family strains and gains, was finally accepted in this study. In other words, the indirect effect of work-family strains and gains on marital satisfaction via parenting stress was observed to be significant.

Next, to validate the indirect effect of parenting stress on the relationship between work-family strains and gains and marital satisfaction, the bootstrapping method suggested by Shrout and Bolger (2002) [50] was used. The bootstrapping method is used to estimate the standard error of the indirect effect that accompanies the previously conducted mediating effect test. Under the method, a confidence interval is presented, and the indirect effect is considered statistically significant when the suggested confidence does not contain 0. As presented in Table 14, the indirect effect of work-family strains on marital satisfaction via parenting stress (β = 0.013, 95% Bias-corrected CI = −0.042∼0.075) and the indirect effect of work-family gains on marital satisfaction via parenting stress (β = 0.013, 95% Bias-corrected CI = −0.042∼0.075) were observed to be statistically significant. That is, marital satisfaction was observed to improve with an increase in parenting stress arising from work-family strains and gains.

## 4. Discussion

### 4.1. Interpretation of the Findings

The present study aimed to validate the mediating effect of parenting stress in the relationship between Korean fathers’ work-family strains and gains and marital satisfaction and identified the importance of parenting stress in the work life and marital life of fathers raising children. The interpretations and implications of this study findings in comparison with those of previous studies are as follows:

First, fathers’ work-family strains were observed to have a positive (+) influence on parenting stress. Considering the findings of a previous study where working mothers reported a higher level of parenting stress than at-home mothers [51], it could be understood that similar results are observed for fathers. This finding is also consistent with another previous finding [52] that fathers who reported experiencing a higher level of work-family strains had a more negative parenting style. In addition, for the specific profession of firefighters, fathers’ work-family strains consistently showed a significant positive association with parenting stress [53]. Based on this, it could be concluded that, similar to mothers, work-family strains also affected parenting stress for fathers. A possible interpretation for such a finding is that for fathers in modern society, the traditional belief that work comes before family in the work-family relationship does not work anymore, and parenting in the family has become increasingly important. Thus, work-family strains affect fathers’ parenting stress due to factors such as time and energy constraints, as well as tensions between different roles, as in the case of mothers.

Second, fathers’ work-family strains were observed to have a negative (−) influence on marital satisfaction. For dual-income couples, serious work-family strains reportedly lead to a poorer quality of marital life [54,55]. However, in the previous study that conducted a more profound investigation of the cause of work-family strains, these researchers examined work-family centrality by dividing couples into husbands and wives [56]. Consequently, they found that marital satisfaction was higher when the husband’s work-family centrality was higher than that of the wife, compared to when the wife’s work-family centrality was higher than that of the husband. This means that the couple’s marital satisfaction level was higher when the husband placed more importance on work than on family compared to when the wife placed more importance on work. In consideration of this, although traditional expectations related to gender roles have been disappearing in modern society, some of those gender role expectations supposedly remain in Asian culture. In this regard, the findings of this study that work-family strains negatively impact marital satisfaction are inconsistent with those of studies in Western countries, where mothers’ work-family strains reportedly led to insufficient time spent with spouses, stress and fatigue, and imbalance in role-sharing, thereby negatively influencing marital satisfaction. Cultural differences could have been an additional factor influencing marital satisfaction.

Third, fathers’ work-family gains did not significantly influence parenting stress. Few studies have been conducted on the relationship between work-family balance and parenting stress for fathers, making it challenging to make a direct comparison. However, extending the scope of comparison revealed that in this study by Cooklin et al. [57], working conditions and job quality in the early stage of parenting positively affected major parenting stages and fathers’ mental health. In this study, the reason why work-family gains did not significantly influence parenting stress was that work-family gains mainly brought about advantages in work life, such as management of working hours or improved work engagement. As for family life, work-family gains were mostly expressed in the form of improved satisfaction by the spouse due to the sharing of housework or parenting duties, indicating that such gains have a direct effect on different environments separately. As parenting stress arises from parenting activities, parenting stress could increase when more time is allocated to parenting with improved work-family balance. Conversely, parenting stress could also decrease as parents become more skilled in parenting through repeated involvement in parenting duties. Thus, more diverse factors must be studied to accurately understand the influencing relationship.

Fourth, fathers’ work-family gains were observed to have a positive (+) influence on marital satisfaction. According to a previous meta-analysis [58], work-family gains could lead to a higher level of engagement, decreased turnover intention, and improved physical and mental health in work life, family life, and everyday life. Considering the findings of individual studies that examined the influence of work-family gains on marital satisfaction, work-family gains could have an indirect positive impact on married couples’ satisfaction by way of wage [59]. Additionally, people might acquire more energy from work-family gains, allowing them to invest more in their spousal roles [60]. However, by making comprehensive reasoning of related factors, we can understand and interpret the findings of this study on the assumption that the father and the mother cooperate on an equal basis to sustain the family and form a peaceful and equal family atmosphere. Presumably, when fathers successfully maintain a balance between work and family, they cooperate more with their spouse and provide more support to them, forming a stable family environment. Such an environment leads to increased marital satisfaction. As mentioned above, this phenomenon has been emerging in the Republic of Korea in recent years, where the culture of gender equity is now more valued than in the past. It is expected to facilitate cooperation between husbands and wives and married couples in showing more respect for each other.

Fifth, fathers’ parenting stress did not have a significant influence on marital satisfaction. While a significantly negative relationship between mother-father conflicts and positive parenting has been reported in previous studies on the effect of marital relationships on parenting [61], most of these studies are limited in that they mainly focused on mothers’ opinions without separately investigating fathers’ positions [62]. Therefore, since those studies were conducted with mothers, not fathers, their findings could have been different from those of this study due to the differences observed between the main and assistant caregivers of the child. In addition, although parenting stress imposes challenges on both the husband and the wife, if the two individuals recognize each other’s roles, share responsibilities, and support each other in the process of overcoming such challenges together, they could develop a mutually supportive relationship. Consequently, parenting stress may not have a negative influence on marital satisfaction. Such findings may have arisen from the general tendency of fathers to distinguish parenting from marriage, viewing the issues related to the child and the spouse as separate.

Finally, for both fathers’ work-family strains and gains, parenting stress was observed to have a partial mediating effect on the relationship with marital satisfaction. It was practically impossible to make a direct comparison with the findings of previous studies, as no previous studies have examined the mediating effect of parenting stress in the relationship between fathers’ work-family balance and marital satisfaction, as set in this research model of this study. However, we could have discussions focusing on studies that have reported some of the relationships among each variable. For instance, Morelra et al. [63] reported in 2019 that work-family strains had an indirect effect on parents’ emotional perception of their child via parenting stress. The authors stated that parents’ ability to concentrate at the moment with their child decreases if they experience a high level of work-family strains and have little time to spend with family. In addition, Wang et al. in 2023 [64] investigated the mediating effect of parenting stress on work-family gains and claimed that parenting stress is affected by work-family gains. They observed that appropriate monetary rewards for work could enable hiring others to care for the family and assist with parenting, thereby reducing parenting stress. Consequently, parents should try to optimize their workplace resources. In another previous study on the mediating effect of parenting stress on married couples’ marital satisfaction [65], parenting stress was observed to mediate the association between the level of infants’ negative emotions and parents’ perceptions of the quality of marriage. These previous findings are in line with those of this study in that in all the previous studies, parenting stress played a mediating role in work-family strains and gains, as well as marital satisfaction.

From the findings of this study, various significant results could be extracted concerning work-family balance for fathers. However, emphasizing the issues that could not be considered in this study, future research is recommended to investigate the following issues:

First, this study was a cross-sectional study on fathers in Korea. However, as public perceptions of the duties and responsibilities socially expected from men in both work and family settings are constantly evolving, a comprehensive analysis is required to identify and understand these changes over time among fathers across diverse cultures.

Second, work-family strains and gains could be experienced in different forms depending on job category, working hours, and the type of employment. Since this study did not account for factors such as perceptions and attitudes toward gender roles, which could impact marital satisfaction and other variables, adopting a new approach considering these additional variables is necessary.

Third, in this study, the survey was conducted under an exceptional situation after the COVID-19 pandemic. Therefore, environmental changes arising from changes in social systems such as social distancing or the spread of remote working could have caused latent bias. For more specific and accurate analysis, future studies need to consider the effects of such exceptional situations as well.

### 4.2. Practical Implications of This Study

This study focused on fathers’ role in the family. As presented above, mainly from the secondary data provided by the PSKC, this study extracted significant results and presented interpretations thereof. Building on those results and interpretations, this study suggests policy implications related to childcare policies for fathers as follows:

First, businesses need to expand the systems to enhance work-family balance. In Korea, currently, there exist institutional benefits for work-family balance for fathers, including paternity leave and flexible working arrangements. However, under the traditional cultural and social atmosphere that mothers should be the main caregivers of the child, such systems have not been widely accepted in reality. Changes in working conditions are essential to expand the role of fathers in the family, and social systems need to be established for that purpose, for instance, by considering imposing legal obligations to make such changes.

Second, direct support needs to be provided to reduce economic burdens for parents. As discussed above, one of the causes of parenting stress in Korea is economic burdens due to people’s eagerness to make huge investments toward their children’s education and the high price tags for private education services. In this context, along with the social systems and benefits for working parents, direct support needs to be offered, even within flexible working environments, to ensure that parents do not suffer economic losses. Indeed, in many advanced countries, economic support for childbirth and childrearing is being provided. The Korean government is also paying childcare allowance, but the amount of money provided each month is USD 80 or so, which cannot be considered sufficient for providing practical help to parents caring for children. Thus, economically practical support must be provided based on accurate calculations of childrearing expenses.

Third, new types of gender equity education are required so that the public can have the right perception of work-family balance for fathers. The role of fathers is rapidly evolving at home and within the family, often at a faster pace than the pace of changes in biases and prejudices concerning gender roles. The need to adjust the role of fathers at work is inevitable to ensure that fathers can take on more responsibilities and play expanded roles in the family. Simultaneously, however, social expectations concerning men’s sacrifice and devotion to work remain high. This causes a new form of conflict between roles, which should be addressed by adopting new types of education. Transforming the public perception through education could result in more work-family gains not only for fathers but also for mothers, and it is highly important for achieving fundamental improvements in the parenting environment.

## 5. Conclusions

This study aimed to validate the structural relationship among Korean fathers’ work-family strains and gains, marital satisfaction, and parenting stress, and the results are as follows:

First, Korean fathers’ work-family strains had a positive (+) influence on parenting stress. Second, Korean fathers’ work-family strains had a negative (−) influence on marital satisfaction. Third, Korean fathers’ work-family gains did not have a significant influence on parenting stress. Fourth, Korean fathers’ work-family gains had a positive (+) influence on marital satisfaction. Fifth, Korean fathers’ parenting stress did not have a significant influence on marital satisfaction. Sixth, in the relationship between Korean fathers’ work-family strains and marital satisfaction, parenting stress had a partial mediating effect. Seventh, in the relationship between Korean fathers’ work-family gains and marital satisfaction, parenting stress had a partial mediating effect.

This study is significant in that it examined fathers’ work-family strains and gains, marital satisfaction, and parenting stress. Additionally, it investigated the relationship among those factors at a time when the global community is facing challenges concerning low fertility rates and numerous family support policies are being implemented across different cultures. This is in line with social changes in today’s world, where the issue of work-family balance is not restricted to mothers and the role of fathers in the family is expanding. The findings of this study are expected to provide a basis for reviewing and implementing new perspectives on family support policies in areas where existing policies have not been effective.

## Figures and Tables

**Table 1:** General Characteristics of this Research Participants.

Variable	Category	Frequency (No. of Persons)	Percentage (%)
Age	In Their 30s (aged 30 or Older and Younger Than 40)	31	2.74
	In Their 40s (aged 40 or Older and Younger Than 50)	879	77.79
	In Their 50s (aged 50 or Older)	220	19.47
Child Sex	Boy	580	51.33
	Girl	550	48.67
Whether They Are Living with The Child	Yes	1072	94.87
	No	58	5.13
Marital Status	Married	1110	98.23
	Single	20	1.77
Educational Background	High School Graduate or with a Lower Level of Education	290	25.66
	Bachelor’s Degree Holder	698	61.77
	Master’s Degree Holder	142	12.57
Total		1130	100

**Table 2:** EFA Results for Work-family Strains.

Category	Item No.	Factor		
		1	2	3
Work-Family Strains	8	0.702	0.238	0.254
	5	0.690	0.254	0.115
	9	0.678	0.161	0.202
	7	0.641	0.317	0.336
	6	0.602	0.316	0.445
	1	0.577	0.141	0.364
	11	0.066	0.763	0.345
	12	0.446	0.724	0.161
	14	0.441	0.713	0.186
	15	0.507	0.711	0.154
	13	0.244	0.666	0.497
	2	0.323	0.161	0.732
	4	0.406	0.264	0.680
	10	0.085	0.485	0.638
	3	0.504	0.274	0.634
Eigenvalue		3.780	3.337	2.820
Covariate (%)		25.199	22.244	18.797
Cumulative Variate (%)		25.199	47.443	66.241
KMO = 0.947, Bartlett’s χ^2^ = 10,402.604 (*p* < 0.001)				

**Table 3:** EFA Results for Work-Family Gains.

Category	Item No.	Factor		
		1	2	3
Work-Family Gains	10	0.835	0.244	0.230
	9	0.752	0.261	0.292
	11	0.752	0.269	0.082
	8	0.739	0.117	0.291
	6	0.246	0.820	0.135
	7	0.331	0.769	0.233
	5	0.234	0.656	0.540
	4	0.235	0.641	0.586
	2	0.269	0.252	0.846
	1	0.319	0.195	0.818
	3	0.185	0.610	0.624
Eigenvalue		2.863	2.792	2.710
Covariate (%)		26.024	25.384	24.640
Cumulative Variate (%)		26.024	51.408	76.048
KMO = 0.915, Bartlett’s χ^2^ = 8655.649 (*p* < 0.001)				

**Table 4:** EFA Results for Parenting Stress.

Category	Item No.	Factor		
		1	2	3
Parenting Stress	1	0.821	0.274	0.102
	3	0.811	0.298	0.140
	2	0.796	0.362	0.049
	4	0.789	0.157	0.342
	5	0.739	0.162	0.366
	6	0.705	0.343	0.267
	11	0.163	0.740	0.290
	9	0.299	0.731	0.166
	10	0.398	0.726	0.009
	16	0.270	0.703	0.156
	12	0.216	0.667	0.424
	7	0.487	0.579	0.238
	14	0.285	0.576	0.456
	15	0.116	0.532	0.248
	17	0.088	0.177	0.772
	8	0.328	0.292	0.688
	13	0.332	0.356	0.599
Eigenvalue		4.582	4.204	2.425
Covariate (%)		26.953	24.732	14.267
Cumulative Variate (%)		26.953	51.684	65.952
KMO = 0.946, Bartlett’s χ^2^ = 12,298.254 (*p* < 0.001)				

**Table 5:** Reliability Analysis.

Variable		Cronbach’s α
Work-Family Gains	Maintaining Identity as an Individual	0.866
	Self-Management	0.881
	Role as a Parent	0.850
Work-Family Strains	Time Constraints	0.850
	Decline in Work Performance	0.831
	Role as a Parent	0.895
Marital Satisfaction		0.935
Parenting Stress	School Variable	0.923
	Education and Learning	0.893
	Aptitude and Growth	0.728

**Table 6:** CFA Model Fit.

Model	χ^2^/DF	RMR	GFI	NFI	IFI	CFI	RMSEA
Hypothesis Model	5.914	0.024	0.954	0.966	0.972	0.972	0.066

**Table 7:** CFA Results.

Variable		Unstandardized Coefficient	S.E.	C.R.	*p*	Standardized Coefficient	Construct Reliability	Average Variance Extracted
Work-Family Strains	→Role as a Parent	1.000	-	-	-	0.863	0.940	0.840
	→Decline in Work Performance	0.902	0.026	34.908	<0.001	0.851		
	→Time Constraints	0.915	0.026	34.832	<0.001	0.850		
Work-Family Gains	→Role as a Parent	1.000	-	-	-	0.697	0.932	0.822
	→Self-Management	1.247	0.048	25.921	<0.001	0.864		
	→Maintaining Identity as an Individual	1.219	0.046	26.255	<0.001	0.900		
Marital Satisfaction	→Spouse	1.000	-	-	-	0.922	0.959	0.853
	→Marital Life	0.990	0.019	52.317	<0.001	0.921		
	→Relationship	1.013	0.021	48.644	<0.001	0.895		
	→Motherhood	0.853	0.023	37.435	<0.001	0.800		
Parenting Stress	→School-related	1.000	-	-	-	0.749	0.886	0.724
	→Education and Learning-related	1.011	0.036	28.189	<0.001	0.930		
	→Aptitude and Growth-related	0.939	0.039	24.316	<0.001	0.728		

**Table 8:** Discriminant Validity.

Variable	Correlation Among Variables				Average Variance Extracted
	Work-Family Strains	Work-Family Gains	Marital Satisfaction	Parenting Stress	
Work-Family Strains	1	-	-	-	0.840
Work-Family Gains	−0.456 ***	1	-	-	0.822
Marital Satisfaction	−0.337 ***	0.430 ***	1	-	0.853
Parenting Stress	0.531 ***	−0.251 ***	−0.192 ***	1	0.724

*** *p* < 0.001.

**Table 9:** Descriptive Statistical Analysis Results.

Variable		Mean	Standard Deviation	Skewness	Kurtosis
Work-Family Strains	Time Constraints	2.33	0.703	−0.037	−0.476
	Decline in Work Performance	2.11	0.692	0.174	−0.499
	Role as a Parent	2.14	0.757	0.118	−0.883
Total		2.19	0.650	0.034	−0.645
Work-Family Gains	Maintaining Identity as an Individual	3.82	0.647	−0.033	0.054
	Self-Management	3.70	0.689	−0.370	1.076
	Role as a Parent	3.84	0.686	−0.348	0.616
Total		3.79	0.595	−0.038	0.075
Marital Satisfaction	Spouse	3.97	0.791	−0.674	0.941
	Marital Life	3.94	0.784	−0.697	1.174
	Relationship	3.90	0.825	−0.682	0.831
	Motherhood	4.02	0.778	−0.746	1.261
Total		3.96	0.727	−0.745	1.611
Parenting Stress	School Variable	2.48	0.879	0.090	−0.829
	Education and Learning	2.32	0.715	−0.074	−0.786
	Aptitude and Growth	3.00	0.849	−0.381	−0.223
Total		2.60	0.713	−0.274	−0.567

**Table 10:** SEM Model Fit.

Model	χ^2^/DF	RMR	GFI	NFI	IFI	CFI	RMSEA
Proposal Model	5.914	0.024	0.954	0.966	0.972	0.972	0.066

**Table 11:** Path Analysis Results.

Path			Standardized Regression Coefficient	Regression Coefficient	S.E.	C.R.	*p*	Tested
Work-Family Strains	**→**	Parenting Stress	0.658	0.663	0.04	16.666	<0.001	Accepted
Work-Family Strains	→	Marital Satisfaction	−0.185	−0.207	0.051	−4.032	<0.001	Accepted
Work-Family Gains	→	Parenting Stress	0.016	0.022	0.045	0.478	0.632	Reject
Work-Family Gains	→	Marital Satisfaction	0.381	0.581	0.056	10.35	<0.001	Accepted
Parenting Stress	→	Marital Satisfaction	0.011	0.012	0.045	0.276	0.783	Reject

**Table 12:** Comparison Between the Hypothesis Model and the Competition Model for Work-Family Strains.

Model	χ^2^	DF	TLI	CFI	RMSEA
Hypothesis Model [Model 1]	186.587	32	0.973	0.981	0.065
Competition Model [Model 2]	261.663	33	0.962	0.972	0.078

**Table 13:** Comparison Between the Hypothesis Model and the Competition Model for Work-Family Gains.

Model	χ^2^	DF	TLI	CFI	RMSEA
Hypothesis Model [Model 3]	139.949	32	0.980	0.986	0.055
Competition Model [Model 4]	321.969	33	0.948	0.962	0.088

**Table 14:** Indirect Effect Analysis.

Path	Estimate	S.E.	Bias-Corrected Bootstrap	
			Lower	Upper
Work-Family Strains → Parenting Stress → Marital Satisfaction	0.013	0.031	−0.042	0.075
Work-Family Gains → Parenting Stress → Marital Satisfaction	0.028	0.012	0.005	0.051

## Data Availability

Available in section “Raw Data” at https://panel.kicce.re.kr/pskc/index.do accessed on 23 September 2024.
